# Key elements of follow-up care after acute pulmonary embolism focusing on long-term sequelae: a Delphi study among European experts

**DOI:** 10.1093/ehjqcco/qcaf053

**Published:** 2025-07-01

**Authors:** Rosa M A Mali, Maarten K Ninaber, Thijs E van Mens, Stavros V Konstantinides, Frederikus A Klok, Delphi Study Contributing Group, George Giannakoulas, Marcin Kurzyna, Marco Zuin, Konstantinos Dimopoulos, Jean Luc Vachiery, Lukas Hobohm, Roberto Badagliacca, Anton Vonk Noordegraaf, Laurent Bertoletti, Luis Jose Jara-Palomares, Olivier Sitbon, Marc Humbert, Silvia Ulrich, Josien van Es, Coen van Kan, Maarten Ninaber, Harm-Jan Bogaard, Esther Nossent, Bas Langeveld, Ivo van der Lee, Ager Andersen, Joanna Pepke Zaba, Dieuwke Luijten, Francis Couturaud, Cecile Tromeur, Olivier Sanchez, Luca Valerio, Menno Huisman, Nick van Es, Cihan Ay, Cecilia Becattini, Irene Lang, Walter Ageno, Melanie Ferreira, Fionnuala Ni Ainle, Roberto Pola, Lilian Meijboom, Lucia Kroft, Karen Sheares

**Affiliations:** Department of Medicine—Thrombosis and Hemostasis, Leiden University Medical Center, LUMC, Room C-7-68, Albinusdreef 2, 2300 RC Leiden, The Netherlands; Department of Pulmonology, Leiden University Medical Center, 2300 RC Leiden, The Netherlands; Department of Medicine—Thrombosis and Hemostasis, Leiden University Medical Center, LUMC, Room C-7-68, Albinusdreef 2, 2300 RC Leiden, The Netherlands; Center for Thrombosis and Hemostasis, University Medical Center, 55131 Mainz, Germany; Department of Medicine—Thrombosis and Hemostasis, Leiden University Medical Center, LUMC, Room C-7-68, Albinusdreef 2, 2300 RC Leiden, The Netherlands; Center for Thrombosis and Hemostasis, University Medical Center, 55131 Mainz, Germany

**Keywords:** Pulmonary embolism, Pulmonary hypertension, Consensus, Echocardiography, Respiratory function tests, Prognosis

## Abstract

**Aims:**

A considerable proportion of patients develop long-term sequelae after an acute pulmonary embolism (PE). Beyond chronic thrombo-embolic pulmonary hypertension (CTEPH), current guidelines provide limited guidance regarding a structured approach for assessment and management of these patients. This study aimed to establish a framework of multidisciplinary follow-up care of PE-survivors.

**Methods and results:**

A Delphi study was conducted among a multidisciplinary panel of PE specialists from across Europe to gather expert opinions, and where possible reach consensus, on key aspects of PE follow-up care. Two rounds of surveys were distributed among 45 venous thromboembolism (VTE) experts, with 39 completing both rounds. Consensus was reached that follow-up of PE survivors should address the entire spectrum of post-PE sequelae, i.e. CTEPH, chronic thromboembolic pulmonary disease, but also all other presentations of the post-PE syndrome. Routine assessment at 3 months should involve patient-reported outcome measures, including quality of life. A single, uniform protocol was preferred over locally adapted approaches. Earlier follow-up, prior to the 3-month mark, to detect post-PE sequelae was not considered necessary for most patient subgroups. Right heart catheterization to confirm CTEPH should be reserved for specialized pulmonary hypertension centres, while other diagnostic modalities such as computed tomography, V/Q scan, cardiopulmonary exercise testing and transthoracic echocardiography can be performed in non-referral centres.

**Conclusion:**

This Delphi study among a panel of VTE experts across Europe describes a consensus-based framework for structured follow-up care for PE-survivors, emphasizing the need for a standardized, multidisciplinary approach to detecting long-term sequelae of PE.

Key Learning Points What is already known:

- A considerable proportion of PE-survivors experience persistent symptoms and functional limitations after an acute PE episode, due to long-term sequelae referred to as post-PE syndrome (PPES).
- Current guidelines are mainly focused on detection of CTEPH but lack comprehensive recommendations for structured follow-up care addressing the full spectrum of these post-PE sequelae.
- There is substantial variability in follow-up care for these patients across, and within, countries.

What this study adds:

- The expert panel advocates for routine evaluation of the presence of the complete spectrum of PPES at 3 months after acute PE diagnosis.
- The expert panel strongly supports integrating patient-reported outcome measures (PROMs), including quality-of-life assessments, into routine follow-up for standardized assessment of long-term post-PE sequelae.
- A uniform follow-up protocol for detection of post-PE sequelae in acute PE survivors is preferred over locally adapted approaches.

## Introduction

Acute pulmonary embolism (PE) occurs frequently and may have potentially life-altering long-term consequences that extend well beyond the acute phase.^1^ During the course of the disease, a broad range of complications may occur in PE survivors, including recurrent venous thromboembolism (VTE), anticoagulant related (major) bleeding and arterial cardiovascular disease.^1-3^ In addition, a substantial proportion of PE survivors are affected by the post-pulmonary embolism syndrome (PPES), characterized by persistent symptoms (i.e. new or persistent dyspnoea, exercise intolerance, cognitive impairment and psychological distress) and functional limitations, despite adequate anticoagulant therapy for at least 3 months and in the absence of an alternative explanation.^3,4^ PPES represents a spectrum of disease states with varying degrees of severity.^5^ CTEPH is the most well-defined and most severe form, though it affects only a small proportion of patients (∼3%).^6,7^ Other presentations of PPES include chronic thromboembolic pulmonary disease (CTEPD), post-PE cardiac impairment and post-PE functional impairment. These long-term sequelae can lead to impaired quality of life, mental health problems and decreased survival (in case of CTEPH), and pose a burden to individual patients and society at large.^3,4,8,9^

Recognition of the need for structured assessment of these long-term sequelae has grown over the past years. The 2019 European guidelines propose an algorithm for detecting post-PE sequelae, with a primary focus on early detection of CTEPH.^10^ This approach leaves a substantial gap in addressing the needs of the larger subset of patients with post-PE functional impairment. A more recently published clinical consensus document by the European Society of Cardiology (ESC) and European Respiratory Society (ERS) advocates standardized evaluation of the presence of persistent symptoms and functional limitations using validated patient reported outcome measures (PROMs).^3^ Despite these recommendations, there is still great variability in follow-up care across European countries possibly due to a lack of convincing evidence regarding the optimal approach to follow-up of PE. Differences in healthcare systems and resources, coupled with the diverse backgrounds of physicians involved in managing PE further contributes to large heterogeneity in follow-up care of PE survivors. This underscores the need for more comprehensive guidance on how to address long-term sequelae to ensure structured follow-up care. The present study aimed to establish a framework of multidisciplinary follow-up care of PE survivors focusing on diagnosing and treating long-term acute PE sequelae.

## Methods

### Delphi technique

The Delphi technique was employed to gather expert opinions among a panel of VTE specialists from across Europe and involving the medical specialties most frequently involved in PE management in order to identify key aspects of follow-up care for patients after an acute PE. This technique is widely used in research to gather expert opinions and reach consensus on topics where empirical data are scarce or unavailable.^11^ It is an anonymous process in which experts are asked to respond to questionnaires. The results of each round are summarized, analyzed and shared as feedback with all participants. Through subsequent surveys, built on the responses of preceding surveys, the aim is to reach consensus.

A three-member multinational steering committee (RM, FK, SK) was formed to guide the process. The initial survey was drafted by FK and RM and provided with feedback by SK. All members approved the final version. The first round of the Delphi study consisted of nine questions which were mostly multiple-choice questions (with options to check multiple answers and always a free text option for comments) or statements with scale of agreement. A final open question was included to allow input from the participants regarding additional relevant topics (see Supplementary material online, *Appendix A* for original survey). Participants were asked to complete the survey within a week. A maximum of two reminders were sent to participants who had not responded after the deadline. After summarizing and evaluating the responses and sharing the feedback from the first round with all the participants, additional rounds were planned to continue until consensus was achieved. Consensus was predefined as ≥70% agreement, anticipating two to three rounds to reach consensus. Google Forms (Alphabet Co., Mountain View, CA) was used to create and distribute the survey.

### Primary outcome

Rather than establishing a new or ‘optimal’ algorithm, this survey focused on more general concepts of follow-up care, including which long-term post-PE sequelae should be routinely evaluated, whether specific patient subgroups should be evaluated sooner than at the standard 3-month mark, or require extended monitoring, and identifying the appropriate setting of follow-up care.

### Selection of experts

Experts were selected based on their expertise in the field of VTE indicated by (i) a relevant publication record and/or leading figures in scientific societies, (ii) clinical experience with diagnosing and/or treating patients with PE and/or membership of the relevant working group or assembly of the ESC and ERS, respectively. Experts were selected to represent a large geographic region (Europe) and a diverse array of specialties, including internal medicine, vascular medicine, angiology, pulmonology, cardiology, haematology and radiology. Throughout the study, participants’ identities and individual responses were anonymous to one another. While FK and RM had access to participant-level data, individual responses were not reviewed, and all analyses were performed at group level. To preserve anonymity, only aggregated, de-identified group feedback was shared with participants after round one.

### Analysis

Descriptive statistics were derived from the results overview in Google Forms.

## Results

The expert panel comprised VTE specialists practising in 14 countries throughout Europe. In total, 51 were invited and 45 agreed to participate. The first round of the Delphi questionnaire was distributed in September 2024, and 41 experts completed the first survey. A total of 39 participants completed the full Delphi procedure. Of the 39 participants who ultimately completed all rounds of the Delphi, 27 (66%) were men, and a broad variety of different specialties was represented including pulmonology (*n* = 18), vascular medicine/angiology (7), cardiology (*n* = 11), radiology (*n* = 2), and haematology (*n* = 2).

### First round

**Figure 1:**
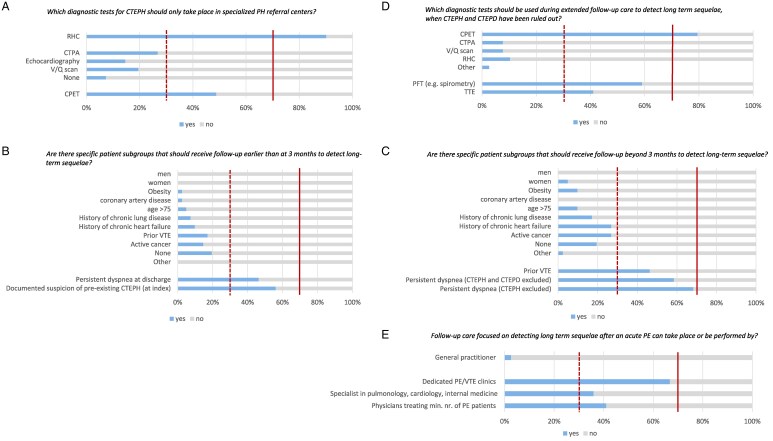
Results from the Delphi. Red dashed lines indicate the threshold of consensus. CTEPH, Chronic thromboembolic pulmonary disease with pulmonary hypertension; CTEPD, chronic thromboembolic pulmonary disease without pulmonary hypertension; PPES, post-pulmonary embolism syndrome (defined as: new onset or progressive dyspnoea, exercise intolerance and/or functional limitations); CPET, cardiopulmonary exercise testing; CTPA, computed tomography pulmonary angiogram; RHC, right heart catheterization; PROMS, patient reported outcome measures.

The first round consisted of nine questions (see Supplementary material online, *Appendix A* for complete survey). Consensus was reached on several topics. The panel agreed that presence of CTEPH (83%), CTEPD (73%) and PPES (90%) all should be routinely evaluated at 3 months after the acute PE diagnosis. Also, the vast majority (90%) agreed that quality of life should be routinely assessed in all patients at 3 months after the index diagnosis. The best way to evaluate quality of life (88% agreed), and simultaneously assess symptom burden and impact of the disease, is by using PROMs. Further, consensus was reached that future guidelines should recommend a single uniform protocol for the follow-up of patients after an acute PE (73% agreed), rather than allowing more flexibility in regionally formed protocols to structure the care pathway based on differences in local resources and healthcare systems. They considered the use of right heart catheterisation (RHC) to confirm CTEPH to be reserved for specialized PH centres (90% agreed), as opposed to other diagnostic tests such as computed tomography pulmonary angiogram (CTPA), V/Q scan, transthoracic echocardiography (TTE), which could also take place in non-specialized PH centres (*Figure 1A*). However, there was no agreement on whether cardiopulmonary exercise testing (CPET) should only be performed in specialized PH centres.

There was consensus that, even in patient subgroups with risk factors for post-PE sequelae (e.g. age >75, gender, obesity, active cancer, history of chronic lung or heart disease, and coronary artery disease), there is no indication for routine testing aimed at identifying long-term sequelae, either before or after the standard 3-month follow-up visit (*Figure 1B* and *C*).

Notably, in patients with prior VTE, the experts agreed that earlier follow-up is not warranted, but no consensus was reached on the need for extended follow-up beyond 3 months in this subgroup of patients. The panel could also not reach agreement on the earlier or extended follow-up in patients with documented suspicion of CTEPH at the moment the index PE was diagnosed or in case of persistent dyspnoea at discharge.

A final area of divergence was the appropriate setting for follow-up care, with 42% of the experts disagreeing on whether this should be centralized in dedicated outpatient clinics vs. performed by general practitioners (GPs) or a more general cardiology/pulmonology department. One of the questions required experts to rate six different aspects of a follow-up algorithm (to detect long-term sequelae) from least relevant/important (6) to most relevant/important (1), allowing each rating number assigned to only one aspect. High sensitivity was considered to be the most important aspect by the majority of the panel (71%), followed by high specificity and cost-effectiveness, which received similar rankings. Avoiding radiation exposure was considered the least relevant by the majority (66%) of the panel. Patient convenience and feasibility in regions or settings without expertise centres were also regarded as less important than high sensitivity, high specificity and cost-effectiveness.

### Second round

The results of the first rounds were summarized and shared with the experts. After evaluating the responses from the first round and incorporating additional input, a second survey was generated. The second survey consisted of eight questions, which were either multiple choice questions (with options to check multiple answers and always a free text option for comments) or statements (see Supplementary material online, *Appendix B* for complete survey). A final open question was added for additional input and comments.

The second round of the Delphi questionnaire was distributed in October 2024 among the 41 experts who had completed the initial survey. The panel was asked to reject or endorse statements regarding earlier (i.e. prior to the 3-month mark) or extended follow-up (i.e. beyond the 3-month mark) in specific patient subgroups to detect long-term post-PE sequelae. They agreed that the follow-up period should be extended in patients with persistent dyspnoea, even when CTEPH (97%) or both CTEPH and CTEPD (77%) are excluded. In a follow-up question, the majority of the panel (79%) considered CPET to be useful as routine diagnostic test in this setting, whereas this was not the case for CTPA, V/Q scan or RHC (*Figure 1D*). No consensus was reached regarding the use of TTE and pulmonary function tests (PFT).

The panel also remained divided on the necessity of subjecting patients with persistent dyspnoea at discharge (46% agreed) or documented suspicion of CTEPH, based on imaging performed at index (41% agreed), to earlier follow-up. The distribution of (dis)agreement regarding earlier follow-up in these patient subgroups was consistent with findings from the first round. Although the majority of the experts disagreed on extending the follow-up period in patients with prior VTE (67%), the threshold for reaching consensus was not reached.

There was consensus against the use of specific diagnostic test or assessments (i.e. CPET, TTE, PFT, CTPA, V/Q scan, RHC) to detect long-term sequelae during the interim period (prior to 3-month mark). The majority of the experts (56%) stated these diagnostics should be considered only after the 3-month follow-up visit since a formal diagnosis of CTEPH and CTEPD can only be made after 3 months of anticoagulation. This aligns with the panel’s consensus against earlier follow-up in most patient subgroups.

With regards to the appropriate setting for follow-up care focused on detecting long-term sequelae after an acute PE, the majority of the experts answered that this ideally is not overseen by a GP (97%). The agreement rate that this should be performed in a dedicated PE/VTE clinic with organized multidisciplinary care was 67%. There were varying opinions on whether follow-up care could be managed by any physician specialized in pulmonology, cardiology, internal medicine or by physicians who treat a minimum number of acute PE patients annually, with agreements rate of 36% and 41%, respectively (*Figure 1E*).

## Discussion

This multidisciplinary Delphi study among VTE experts across Europe addresses several key aspects of follow-up care focused on detecting long-term sequelae of PE. These findings may form the framework for a structured, dedicated follow-up care for PE survivors in the European setting. The most notable findings of this study include the consensus that (i) follow-up of PE survivors should encompass the entire spectrum of post-PE sequelae rather than focusing solely on early detection of CTEPH or CTEPD, (ii) PROMS including, measurement of quality of life, play a central role, (iii) diagnostic accuracy is considered more important than patient convenience and (iv) a single, uniform protocol is preferred over local translation of a more generalized approach. Developments in post-VTE care and expanding therapeutic options such as balloon pulmonary angioplasty, pulmonary endarterectomy and (structured) rehabilitation programmes reinforce the need for a structured approach to PE follow-up and routine assessment of the complete picture of outcomes of patient care.^9,12-16^

Despite multiple algorithms and guidelines available for CTEPH detection and management, diagnostic and therapeutic approaches for PPES in general remain less well-defined. While CTEPH is a severe but relatively rare condition,^7^ PPES is far more common, affecting up to 50% of patients.^4,17^ Patients may experience symptoms such as fatigue, pain, and anxiety, which can profoundly impact their quality of life but are not captured by diagnostic tools in current algorithms, such as echocardiography or V/Q scans. CPET has been proposed as a useful diagnostic tool for evaluating persistent symptoms, when CTEPH and CTEPD have been ruled out.^18^ Our study supports the recommendations of the ESC position paper, emphasizing the need for a comprehensive approach that addresses the full spectrum of post-PE sequelae, including the role of CPET in symptom evaluation. Even so, an important part of the post-PE syndrome are psychosocial complications such as anxiety and depression.^19-23^ These are not captured by routine history taking, echocardiography or exercise tests, but require targeted questioning of the patients and/or questionnaires facilitating self-reporting of such complications.

PROMs have emerged as effective tools for capturing the impact of PPES, providing a comprehensive assessment of symptoms, functional limitations, and quality of life.^24-26^ Their routine integration into clinical practice facilitates systematic monitoring of post-PE recovery, enabling early identification of patients at risk for the full spectrum of post-PE sequelae, guiding subsequent diagnostic evaluation and (possible) therapeutic interventions. In addition, PROMs contribute to evaluating treatment efficacy and optimising resource allocation. While a single PROM was already included in the 2019 ESC/ERS guideline, their use was strongly endorsed in the more recent ESC position paper.^3,10^ A standardized set of core patient centred outcomes has been proposed recently, in which PROMs are positioned as a central element of structured PE follow-up.^26^ Among the proposed instruments are questionnaires to capture dyspnoea, mood and pain, but also quality of life. The latter was identified in this study as a relevant part of routine follow-up of acute PE. Notably, explicit measurement of quality of life is currently not yet a standard component of PE follow-up. Quality of life is multifaceted and influenced by various factors. Although clinically relevant differences for PE specific quality of life tools have been established,^27^ it may be challenging to intuitively use the measure in daily clinical practice. Nonetheless, integration of PROMs in the patient care pathway aligns with the changing perspective on outcome assessment in medicine, focusing more on patient defined outcome measurements leading to more patient centred healthcare.^28^ Importantly, implementation of PROMs in clinical practice poses challenges, including, but not limited to, adherence from both patients and physicians and access of appropriate digital infrastructure to ensure integration into existing healthcare systems. Robust ICT support will be crucial for the successful and routine use of PROMs in clinical settings.

One of the questions to the expert panel was whether a rigid, algorithm-based approach should be adopted, or if a more flexible model with clear definitions of follow-up criteria and outcomes but adapted to local preferences and resources should guide follow-up care. The current ESC guidelines advocate for an integrated approach tailored to individual healthcare system capacities, but still propose a clear stepwise approach. Results from our study confirmed the preference for a single, uniform protocol. A standardized algorithm may indeed improve the current inconsistencies in follow-up care, although flexibility may be necessary to facilitate implementation based on local differences in healthcare systems and responsible physicians. Several follow-up algorithms have been studied in recent years, each proposing different approaches to balancing feasibility, cost-effectiveness, and clinical effectiveness. A recent cost-effectiveness analysis highlights that while different follow-up strategies exist, their overall efficiency and related cost-effectiveness remain relatively comparable.^29^ Feasibility of wide implementation of any standardized follow-up programme likely varies between countries and healthcare settings and is largely dependent on which healthcare professionals mainly are responsible for the after care of PE patients, and the availability of resources for diagnostic testing. Experts in our panel considered sensitivity and specificity to be the most important aspect of the follow-up algorithm, followed by cost-effectiveness. This is in line with current algorithms for early CTEPH detection, since diagnostic delay is associated with worse outcomes including mortality.^30^

The setting in which follow-up care is provided plays a crucial role. Our panel did not reach a consensus on whether follow-up should take place primarily in specialized PE/PH centres with active EXPERT-PE teams, in (more) general hospitals, or be managed by physicians treating a minimum number of acute PE patients, annually. While our panel expressed a clear preference against follow-up care focused on long-term sequelae being managed by a GP, this reflects the perspectives of the panel members, all of whom are hospital-based medical specialists. GPs were not represented in this study. Given the current lack of standardized protocols for follow-up with regards to long-term sequelae, and the limited access in primary care to diagnostic tools (such as echocardiography and CPET), it seems reasonable that this responsibility should not be placed with primary care physicians.

At the same time, it is unrealistic that all PE patients can be managed in tertiary care centres. Instead, a more viable solution may lie in a structured approach that equips local healthcare providers with the necessary knowledge, resources and referral pathways.

This study included a broad range of VTE experts across Europe, representing all the medical specialties most frequently involved in the management and follow-up after acute PE including both clinically oriented physicians and leading clinical researchers in the field of VTE. The high participation and response rate strengthen the validity of the consensus that was reached on key aspects. Certain limitations should nonetheless be acknowledged. This study had an exploratory nature, aiming to address key aspects rather than establish definitive consensus. The findings represent, therefore, expert opinion and definitely not evidence-based recommendations. Its value lies in highlighting the current agreement among experts. While our findings reflect expert consensus, they underscore the need for prospective studies to validate the proposed framework—particularly regarding the clinical effectiveness, cost-effectiveness, and feasibility of structured follow-up protocols for PE survivors. A recent Danish research project has developed, implemented and evaluated feasibility and cost-effectiveness of a structured follow-up programme for PE survivors. Although its primary focus was not specifically on long-term sequelae, the study provides valuable insight into the feasibility and potential benefits of a systematic post-PE follow-up approach in routine clinical practice.^31-33^

In conclusion, this Delphi study provides insights into current expert consensus on organization and focus of post-PE follow-up care, providing a contemporary framework that may serve as inspiration for further research as well as for future guidelines.

## Supplementary Material

qcaf053_Supplementary_Data

## Data Availability

The data underlying this article will be shared on reasonable request to the corresponding author.
