# Supplementary material for: Key elements of follow-up care after acute pulmonary embolism focusing on long-term sequelae: a Delphi study among European experts
Source: Eur Heart J Qual Care Clin Outcomes. 2025 Jul 1;11(7):1137–43. doi: 10.1093/ehjqcco/qcaf053 (PMC12587279; doi:10.1093/ehjqcco/qcaf053)
Supplement: qcaf053_Supplementary_Data [file qcaf053_supplementary_data.docx]

## Appendix

**Appendix A.** Questions included in first round of Delphi survey.

1. What long-term pulmonary embolism (PE) sequelae should be routinely evaluated at 3 months after an acute PE diagnosis?

- Chronic thromboembolic pulmonary hypertension (CTEPH)
- Chronic thromboembolic pulmonary disease without pulmonary hypertension (CTEPD)
- Post-pulmonary embolism syndrome (defined as: new onset or progressive dyspnea, exercise intolerance and/or functional limitations
- Other

2. Are there specific patient subgroups that should receive follow-up earlier than at 3 months to detect long-term PE sequelae?

- Age > 75
- Women
- Men
- Obesity
- Active Cancer
- Prior VTE
- Patients with documented suspicion of pre-existing CTEPH
- History of chronic heart failure
- Coronary artery disease
- History of chronic lung disease
- Patients with persistent dyspnea at discharge
- None
- Other

1. In which order would you rank the following aspects of a screening algorithm for long-term sequelae in PE survivors from most (6) relevant/important to least (1) relevant/important)?
   - Cost-efficacy
   - High sensitivity
   - High specificity
   - Feasibility in regions/setting without expertise centers
   - Convenience for patients
   - Avoiding radiation exposure

4. Would you agree with the following statement: Quality of life should be assessed in all patients at 3 months after an acute PE.

- Strongly agree
- Agree
- Disagree
- Strongly disagree

5. Would you agree with the following statement: Patients reported outcome measures (PROMs) should be routinely used to assess symptom burden and impact of the disease at 3 months after an acute PE.

- Strongly agree
- Agree
- Disagree
- Strongly disagree

6. Would you agree with the following statement: Future guidelines should recommend a single, uniform protocol for the follow-up of patients after an acute PE.

- Strongly agree
- Agree
- Disagree
- Strongly disagree

7. Are there specific patient subgroups that should receive follow-up earlier than at 3 months to detect long-term PE sequelae?

- Age > 75
- Women
- Men
- Obesity
- Active Cancer
- Prior VTE
- History of chronic heart failure
- Coronary artery disease
- History of chronic lung disease
- Patients with persistent dyspnea in whom CTEPH has been excluded
- Patients with persistent dyspnea in whom CTEPH and CTEPD have been excluded
- Other

8. Should follow-up after acute PE be centralized in dedicated outpatient clinics (alternative could be follow-up by general practitioners or a more general cardiology/pulmonology department)?

- Yes
- No

9. Which screening diagnostics for CTEPH should only take place in specialized PH centers?

- CT angiography of the chest (CTPA chest)
- V/Q scan
- Echocardiography
- Right heart catheterization
- Cardiopulmonary exercise testing
- None
- Other

10. What additional insights or considerations do you believe are important concerning the follow-up care of patients after an acute PE that have not been addressed in this questionnaire? (Free text)

**Appendix B.** Questions included in second round of Delphi survey.

1. Patients with a documented suspicion of CTEPH (based on imaging performed at index) should receive follow-up earlier than at 3 months after an acute PE diagnosis to detect long-term post-PE sequelae.

- Agree
- Disagree

2. Patients with persistent dyspnea at discharge should receive follow-up earlier than at 3 months after an acute PE diagnosis to detect long-term post-PE sequelae.

- Agree
- Disagree

3. In light of the fact that a formal diagnosis of CTEPH and CTEPD can only be made after 3 months of anticoagulation, what diagnostic tests or assessments would you use in the interim (prior to 3-month mark) for specific subgroups that in your opinion should receive follow-up earlier than at 3 months to detect long-term post-PE sequelae?

- Cardiopulmonary exercise testing (CPET)
- Transthoracic echocardiography (TTE)
- Pulmonary functions test (*e.g.,* spirometry, diffusion capacity)
- CTPA
- V/Q scan
- Right heart catheterization (RHC)
- None (this should wait until the 3-month follow-up visit unless overt right heart failure is present

4. Patients with persistent dyspnea in whom CTEPH is excluded, should receive extended follow-up beyond 3 months do detect long term sequelae.

- Agree
- Disagree

5. Patients with persistent dyspnea in whom CTEPH and CTEPD is excluded should receive extended follow-up beyond 3 months do detect long term sequelae.

- Agree
- Disagree

6. Patients with prior VTE should receive extended follow-up beyond 3 months do detect long term sequelae.

- Agree
- Disagree

7. What diagnostic tests or assessments would you use/propose for specific patient subgroups that in your opinion should receive extended follow-up care beyond 3 months to detect long term PE sequelae, if CTEPH and/or CTEPD have been ruled out.

- Cardiopulmonary exercise testing (CPET)
- Transthoracic echocardiography (TTE)
- Pulmonary functions test (*e.g*., spirometry, diffusion capacity)
- CTPA
- V/Q scan
- Right heart catheterization (RHC)
- None

8. Follow-up care focused on detecting long term sequelae after an acute PE can take place or be performed by:

- Any general practitioner
- Any physician specialized in pulmonology, cardiology or internal medicine
- Physicians who treat a minimum number of acute pulmonary embolism patients per year (*e.g.,* 5 /year)
- Dedicated PE/VTE clinics with organized multidisciplinary care
- Other

9. Do you have any additional insights or considerations that you believe are important concerning the follow-up care of patients after an acute PE that have not been addressed in this questionnaire? (Free text)
